# Cerebral aspergillosis after heart-lung transplantation in a child: Case report with 3-year follow-up and literature review

**DOI:** 10.3389/fcvm.2022.1042631

**Published:** 2023-01-06

**Authors:** Huanwei Zhuang, Kun Xiang, Shuji Gong, Yangang Zhou, Jinlan Chen

**Affiliations:** ^1^Department of Cardiovascular Surgery, The Second Xiangya Hospital, Central South University, Changsha, China; ^2^Department of Pharmacy, The Second Xiangya Hospital, Central South University, Changsha, China; ^3^Department of Cardiac Surgery, Haikou Affiliated Hospital of Central South University Xiangya School of Medicine, Haikou, China

**Keywords:** heart-lung transplantation, intracranial infection, *Aspergillus*, metagenomic next-generation sequencing (mNGS), voriconazole

## Abstract

There are limited cases of heart-lung transplantation (HLT) in children worldwide owing to lack of donors, demanding surgical teamwork, and arduous post-operative management. Post-transplant management difficulties stem from the possible development of several post-operative complications, with infection being a common complication. Intracranial fungal infections are difficult to diagnose and prone to treatment delays because of their relatively insidious onset and atypical clinical presentation. Here, we present a case of a cerebral infection developed 3 months after HLT in a 10-year-old child, showing no positive results on conventional imaging or cerebrospinal fluid (CSF) examination and culture. On metagenomic next-generation sequencing of the cerebrospinal fluid, the causative organism was finally determined as *Aspergillus*. After administering 1-year anti-*Aspergillus* treatment, no recurrence of intracranial fungal infection was noted during the 3-year follow-up. This case illustrates the multifaceted diagnostic techniques for cerebral aspergillosis after HLT and shows the significance of dynamic monitoring of symptoms, such as headache, and of metagenomic sequencing results, trends in intracranial pressure and (1-3)-β-D-glucan levels for guiding diagnosis and treatment.

## 1. Introduction

Heart–lung transplantation (HLT) is the only treatment for certain end-stage cardiopulmonary diseases so far. In 1968, Professor Cooley performed the first human HLT on a 2-month-old infant, who died of pulmonary insufficiency 14 h after the procedure (1). In recent years, the short- or long-term results of HLT have become increasingly more satisfactory due to improvements in donor organ preservation, surgical techniques, and postoperative management as well as advancements in immunosuppressive treatment. However, post-transplant rejection, infections, renal failure, tumors, and other postoperative complications continue to occur and adversely affect quality of life. Infections have become the most prevalent complication due to the administration of immunosuppressive drugs after transplantation. The most common site of infection is the lungs, but intracranial infections are the most insidious and are more challenging to diagnose and treat. Common pathogens that cause infection include bacteria, viruses, and fungi. Compared to invasion by bacteria and viruses, intracranial fungal infections are less common, with *Candida* spp. and *Aspergillus* spp. being predominant (2). However, diagnosing and treating intracranial fungal infections remain challenging in clinical neurology, and routine CSF examination and culture are often negative. The early application of metagenomic next-generation sequencing (mNGS) can help identify the pathogenic organism, ensuring the provision of appropriate treatment. The treatment of post-transplantation intracranial fungal infection includes reduction of immunosuppressive drugs, commencement of standardized medication, and long treatment courses. The monitoring of trends in symptoms, intracranial pressure, mNGS, and biochemical indicators in the CSF can help guide treatment and achieve more favorable outcomes.

## 2. Case description

Our patient was a 10-year-old girl with complex congenital heart disease identified at birth. Congenital heart malformations included single atrium, single ventricle (functionally a right ventricle), pulmonary artery stenosis, and a persistent left superior vena cava. The child underwent bidirectional Glenn shunt operation and total cavopulmonary connection in March 2010 and June 2013, respectively. Due to end-stage cardiopulmonary failure, the patient finally underwent *in situ* HLT in April 2019. The child was extremely unstable and needed extracorporeal membrane oxygenation (ECMO) when back to ICU from the operating theater, and suffered from functional impairment of multiple organs. When the clinical picture improved, she had bacterial pneumonia and viral septicemia. With the provision of support and protection of organ function as well as the adjustment of immunosuppressants and treatment with anti-infectives, the child fully recovered 2 months later. At 3 months postoperatively, the patient occasionally experienced self-resolving headaches. At that time, intracranial calcification was seen on cranial computed tomography with no other abnormalities. Two weeks later, the patient’s headache worsened. A lumbar puncture revealed a CSF pressure of 380 mmH_2_O. The CSF tested positive on Pan’s test, but negative for *Cryptococcus neoformans* capsular antigen and ink staining, and negative on culture. Accordingly, a preliminary clinical diagnosis of intracranial infection was made.

**FIGURE 1:**
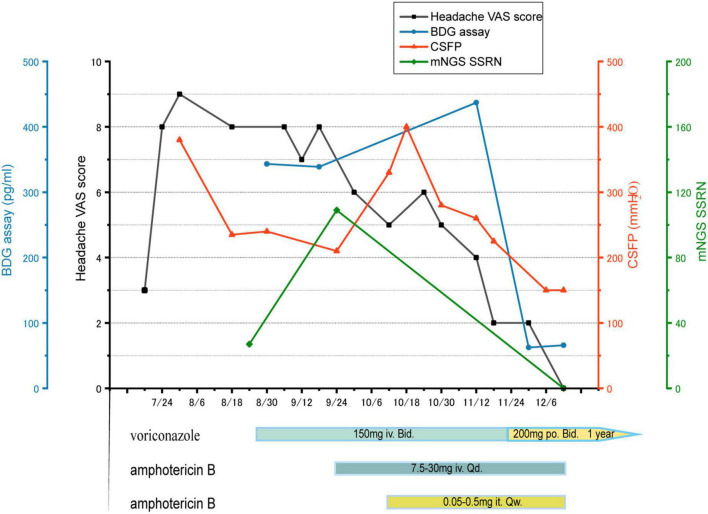
Headache VAS score (black), BDG assay (blue), CSFP (orange), mNGS SSRN (green). The treatment course: voriconazole iv for 4 weeks, but both of mNGS SSRN and CSFP increase, so intrathecal and intravenous amphotericin B were added, combined with voriconazole, for 8 weeks. Then voriconazole po for a year. VAS: visual analogue scale, BDG: (1-3)-β-D-glucan, CSFP: cerebrospinal fluid pressure, mNGS: metagenomic next-generation sequencing, SSRN: species-specific read number, iv: intravenous injection, po: *per os*, it: intrathecal injection.

Further cranial plain magnetic resonance imaging with enhancement, magnetic resonance angiography, and magnetic resonance venography revealed no significant abnormalities. During this period, repeated CSF culture was negative. But CSF manometry showed high levels, and CSF (1-3)-β-D-glucan was positive through G test, which suggested a high probability of an intracranial fungal infection. However, the causative organism was not identified. Testing of the CSF using mNGS revealed a high sequence number relating to *Aspergillus* species (mainly *Aspergillus niger*), thus confirming the diagnosis of cerebral aspergillosis. Regarding treatment, the prednisolone tablets were discontinued to reduce immunosuppression, and the antifungal voriconazole was given. After 4 weeks of voriconazole treatment, the child’s headache symptoms did not resolve. There was no significant decrease in CSF pressure, and the patient had persistent positive G test results. A repeated mNGS check showed an increased sequence number of *A. niger*. As monotherapy was deemed ineffective, intravenous amphotericin B was added with supplemental intrathecal administration. After 8 weeks of treatment with voriconazole and amphotericin B, the child’s headache gradually resolved and subsequently disappeared. The intracranial pressure gradually decreased to normal, the G test result turned negative, and repeated mGNS tests of the CSF became negative (see Figure 1 for the specific treatment course). After a 12-week induction period, the amphotericin B was discontinued and maintenance therapy with oral voriconazole alone was administered for 1 year. At the end of the 3-year follow-up, the child had experienced no recurrence of the intracranial infection.

## 3. Discussion

With recent advancements in HLT and postoperative management, patient survival rates have improved significantly. The current 3-month, 1-year, 3-year, 5-year, and 10-year survival rates are 71, 63, 51, 44, and 31%, respectively (3). More complications are likely to occur associated with these higher survival rates. Possible graft-related complications include early graft dysfunction, acute allograft rejection, and heart graft vascular disease, while possible non-graft-related complications include infection, acute or chronic kidney injury, and malignancy (4).

The post-operative management of HLT in children poses great difficulty. Repeated preoperative cardiopulmonary failure may compromise extracardiac organs, including those that mediate immune function. This involvement, combined with the use of post-operative immunosuppressive agents, makes patients highly susceptible to infection. Postoperative infections that occur after solid organ transplantation are typically divided into three stages. Nosocomial infections comprise the majority of them in the early postoperative period, followed by opportunistic infections in the mid postoperative period and community-acquired infections in the late postoperative period (5). Pulmonary infection is the most common nosocomial infection, followed by urinary tract, hematologic, incisional, and intracranial infections. Intracranial infection is also more common after transplantation, and the responsible organisms are primarily bacteria (including even *Mycobacterium tuberculosis*), viruses, and fungi. Fungi that cause intracranial infections are invasive, the most common being caused by *Candida*, then *Aspergillus*, and other genera of fungi such as *Cryptococcus* (6–8). *Aspergillus* is a common causative organism of mycotic infections in humans, and most cases of cerebral aspergillosis are caused by *Aspergillus fumigatus* (6, 8).

Alexander et al. found additional induction immunosuppression, reoperation within 10 days of heart transplant, delayed chest closure and peri-transplant ventricular assist device (VAD) placement were associated with an increased risk of invasive fungal disease (IFD) (9). The risk factors for central nervous system (CNS) aspergillosis include neutropenia, systemic glucocorticoid treatment, mastoidectomy, spinal anesthesia, and paraspinal glucocorticoid injections (10). And risk factors for the development of intracranial fungal infections within 3 months post-transplantation include the use of vasoactive drugs, extended intensive care unit stay, renal failure requiring hemodialysis, and bacterial infection (11). In immunocompromised patients, the fungus often reaches the bloodstream through the respiratory tract and may disseminate to spread to the brain. Intracranial infection may also be caused by the fungus spreading through adjacent structures such as the sinuses, orbits, and mastoid process (12). Intracranial fungal infections in children are uncommon but can be fatal; hence, their early diagnosis and treatment are crucial.

Intracranial fungal infections can present as various clinical syndromes such as meningitis, encephalitis, brain abscess, and even rarely as stroke (7, 13). Clinical symptoms include nausea, vomiting, headache, fever, confusion, and seizures (6, 14), but all are non-specific. Procedures such as microscopic examination, CSF culture, and histopathological examination of fungal pathogens constitute the gold standard for diagnosis (8, 15). However, CSF culture is time-consuming and has a low sensitivity, and specimens are often difficult to obtain for pathological examinations; thus, CSF analysis and imaging remain the basis for diagnosis. The presence of elevated pressure and protein levels along with decreased glucose levels in the CSF suggest an intracranial infection (13). These markers are important adjuncts to computed tomography and magnetic resonance imaging for the detection of infection and the monitoring of treatment.

Additional antigen analyses have been used to diagnose cerebral aspergillosis and monitor treatment efficacy. The substance (1,3)-β-D-glucan, which is detected using the G test, is a conserved component of the fungal cell wall. The test is indicated for the early diagnosis of all deep fungal infections except those caused by *Cryptococcus* and *Trichophyton*, but not for determining fungal infection type. The other substance of note is galactomannan detected using the GM test, which is primarily targeted for the early diagnosis of invasive aspergillosis. Its use is recommended by the Infectious Diseases Society of America for diagnosing aspergillosis. Polymerase chain reaction (PCR) of fungal-specific DNA is useful for diagnosing mycobacterial infections, but the results should be considered in conjunction with those of other tests and the clinical context (16). The current case showed only a strongly positive G test result of the CSF, suggesting an intracranial fungal infection. However, the child’s GM test was negative. As infections caused by *Aspergillus* are often accompanied by a positive GM test, it was possible that antifungal therapy led to a false negative GM test result in this case. However, it is difficult for the conventional tests mentioned above to determine the causative organism; thus, more advanced testing techniques are needed.

The mNGS technology is a DNA sequencing technique that was developed from PCR and gene chips and belongs to a group of modern molecular diagnostic techniques. Since the first reported case of leptospirosis diagnosis using mNGS in 2014, mNGS has rapidly become a complementary test for various infections. Compared to traditional tests, mNGS is more effective at identifying CNS infections, with a positive rate of 57% (17). The combination of mNGS with traditional microbiological assays can significantly increase positive rates, especially in cases with difficult-to-grow fungi and low fungal culture loads. In mNGS detection, the thresholds of species-specific read number (SSRN) of different genera showing a positive result are inconsistent, with an SSRN ≥2 showing positive results for *Aspergillus*. In this case, the SSRN in the first mNGS test for *Aspergillus* was 27 and *A. niger* was 20, thus confirming its identity as the causative organism. During the treatment with voriconazole, mNGS was tested again and the SSRN of *A. niger* detected in the CSF reached 109. The increase in sequence number implied that the treatment was sub-optimal if not ineffective; thus, it was intensified by the addition of amphotericin B. At the end of the induction phase, no pathogenic organisms were detected during the final test, which shows the significance of mNGS for guiding treatment.

The diagnosis of cerebral aspergillosis is challenging, with only 55.9% of patients being diagnosed while still alive (10). The disease also has poor prognosis and high mortality rates. A review shows that Aspergillus meningitis has an ominous prognosis with a global case-fatality rate (CFR) of 72.1% but with a much better outcome among immunocompetent patients in whom a CFR of 63.5% was observed versus a 83% CFR registered among immunocompromised patients (10). So once diagnosed, a standardized and complete pharmacological treatment course is essential. Triazoles, particularly voriconazole, isavuconazole and posaconazole for invasive infections, and voriconazole or itraconazole for chronic infections, are the first line antifungal agents used to treat aspergillosis (18). The availability of both intravenous and different oral formulations of triazoles increases the therapeutic options. Voriconazole is the current treatment of choice for cerebral aspergillosis, while amphotericin B is reserved for voriconazole-intolerant or -refractory patients. The combination of the two is used as primary or remedial therapy for refractory aspergillosis. Once the induction period is over and the disease stabilizes, oral voriconazole may be administered for a year. In the current case, voriconazole administered intravenously for 4 weeks was ineffective; the child’s headache was not relieved, the microorganisms persisted, and the intracranial pressure did not decrease. Hence, intrathecal and intravenous amphotericin B was added, starting at a low dose and gradually increasing to a maintenance dose. After 8 weeks of the above combination treatment, all the patient’s symptoms and test results returned to normal; hence, the amphotericin B was discontinued and oral voriconazole was commenced. The patient experienced severe hypokalemia while receiving the amphotericin B. Daily monitoring and supplementation ensured safe treatment, and this case demonstrates the need for close monitoring of adverse effects during amphotericin B administration.

## 4. Conclusion

Cerebral aspergillosis, which is relatively rare after HLT, is clinically difficult to diagnose due to its insidious onset and low sensitivity of traditional detection methods. In addition to traditional detection methods, mNGS is recommended as early as possible as an adjunct for identifying the pathogenic organism. Once the diagnosis is confirmed, a standardized and long course of antifungal treatment should be administered. The findings in this case showed that cerebral aspergillosis after transplantation in children is difficult to treat, as it requires combination voriconazole and amphotericin B and a long induction treatment period to achieve clearance of the microbial load. Closely observing the patient’s clinical symptoms, using the G-test, and monitoring the intracranial pressure and mNGS during treatment may guide treatment and achieve satisfactory outcomes.

## Data availability statement

The original contributions presented in this study are included in the article/supplementary material, further inquiries can be directed to the corresponding author.

## Ethics statement

Written informed consent was obtained from the minor(s)’ legal guardian/next of kin for the publication of any potentially identifiable images or data included in this article.

## Author contributions

HZ and JC contributed to the conception and design of the study. HZ completed the data collection and wrote the first draft of the manuscript. HZ, JC, KX, SG, and YZ wrote the sections of the manuscript. All authors contributed to manuscript revision, read, and approved the submitted version.
